# Preparing future physicians for complexity: a post-graduate elective in HIV psychiatry

**DOI:** 10.1186/s12909-023-04233-0

**Published:** 2023-04-20

**Authors:** Deanna Chaukos, Sandalia Genus, Robert Maunder, Maria Mylopoulos

**Affiliations:** 1 Department of Psychiatry, Temerty Faculty of Medicine, University of Toronto, Toronto, Canada; 2 Department of Psychiatry, Sinai Health System, 600 University Avenue, Toronto, M5G1X5 Canada; 3 Department of Anthropology, University of Toronto, Toronto, Canada; 4 Department of Pediatrics, Temerty Faculty of Medicine, University of Toronto, Toronto, Canada; 5 The Wilson Centre, University of Toronto, Toronto, Canada

**Keywords:** Resident education, Psychiatry, Complexity, Collaborative and team-based care, Interprofessional learning, Adaptive expertise

## Abstract

**Background:**

Patients with complex care needs have multiple concurrent conditions (medical, psychiatric, social vulnerability or functional impairment), interfering with achieving desired health outcomes. Their care often requires coordination and integration of services across hospital and community settings. Physicians feel ill-equipped and unsupported to navigate uncertainty and ambiguity caused by multiple problems. A HIV Psychiatry resident elective was designed to support acquisition of integrated competencies to navigate uncertainty and disjointed systems of care – necessary for complex patient care.

**Methods:**

Through qualitative thematic analysis of pre- and post-interviews with 12 participants – residents and clinic staff – from December 2019 to September 2022, we explored experiences of this elective.

**Results:**

This educational experience helped trainees expand their understanding of what makes patients complex. Teachers and trainees emphasize the importance of an approach to “not knowing” and utilizing integrative competencies for navigating uncertainty. Through perspective exchange and collaboration, trainees showed evidence of adaptive expertise: the ability to improvise while drawing on past knowledge.

**Conclusions:**

Postgraduate training experiences should be designed to facilitate skills for caring for complex patients. These skills help residents fill in practice gaps, improvise when standardization fails, and develop adaptive expertise. Going forward, findings will be used to inform this ongoing elective.

## Background

Patients with complex health needs are described as having multiple, concurrent conditions, including mental illness, social vulnerability, or functional impairment which interfere with achieving desired health outcomes [1–3]. These patients may present atypically, with multiple possible etiologies, and often require personalized care that is time and resource intensive. This requires the coordination and integration of services across hospital and community-based settings [4–8]. Frequently this care might deviate from best practice recommendations and the ideal of standardization, which is seen as one way to improve quality and contain costs [9]. Thus, physicians often find themselves working to navigate ambiguity caused by multiple medical, psychiatric or psychosocial etiologies within the constraints of a system that is designed to deliver standardized care. As a result, physicians feel-ill equipped and unsupported to care for these patients [3, 10].

Because patients often have complex health needs, it is important to develop and support physicians to work with medically and psychiatrically complex patient populations, like those with HIV and mental illness [3, 11, 12]. Residents exiting advanced stages of training should have the knowledge, skills and attitudes to address complexity, and this learning should not be serendipitous, based on rotation or supervisor. Drawing from an adaptive expertise conceptual framework, developing an approach to complexity requires trainees to expand their capacity to integrate contextual knowledge about a patient (for example: their psychosocial history, medical and psychiatric history) as well as different (perhaps conflicting) perspectives on their care [8]. Adaptive experts are able to efficiently utilize known solutions when appropriate, while also improvising an evidence-informed approach when “knowing” is not possible. [13]. Yet many required training experiences fail to recognize the specific challenges and capabilities required for caring for complex patients.

Moreover, the reality of our health care system requires physicians to work collaboratively as part of teams, including informal teams that span hospital and community settings [10, 13–15]. Though CanMEDS [16] recognizes the importance of the diverse roles of physicians (collaborator, communicator, advocate, medical expert, etc.), curricula in medical education under-recognize the required integration of these roles to achieve key competencies that underpin collaborative practice – like shared decision making, problem solving, or collaborative learning [17, 18]. Adaptive expert capabilities emphasize integrative competencies that combine medical expertise with collaborator roles, often across hospital and community settings, and amidst scarce resources.

To address these gaps, we designed a senior resident elective experience in HIV Psychiatry that specifically aims to prepare physicians for work with complex patient populations through the development of adaptive expertise. The elective was developed four years ago and is based on years of collective teaching experiences in HIV Psychiatry at the intersection of Psychiatry, Infectious Disease, and community agencies. Learning objectives were developed from a needs assessment which included diverse stakeholder perspectives: people with lived experience of HIV, community workers and hospital-based clinicians [18]. Here we describe the results of a qualitative study using thematic analysis of interviews with residents and clinic staff conducted before and after the HIV Psychiatry elective experience.

## Methods

### Context

This study is part of a broader educational research initiative that aims to improve coordination of care between hospital and community for patients with HIV and mental illness who receive care through the Clinic for HIV-related Concerns in the Department of Psychiatry at Sinai Health, Toronto. Established in 1986, the clinic provides mental health services for people with HIV, mental illness and often other intersectional factors and social determinants of health impacting access to care. The interprofessional team includes psychiatrists, an occupational therapist, and a social worker. Services include psychiatric assessment and management of major psychiatric disorders, including psychotherapy. The clinic hosts trainees, such as medical students, non-MD clinicians, and residents in psychiatry and other specialities.

### Intervention

Residents participating in the elective are embedded as part of the interprofessional team in the clinic, and care for complex patients with HIV, mental illness, other medical problems, and psychosocial challenges (frequently trauma). Often the patients are challenging to engage in treatment, have multiple comorbid medical and psychiatric problems, and the referring team is uncertain how to proceed with their care. The aim of this resident elective is to enhance skills for care of complex patients, equipping future psychiatrists to fill in practice and knowledge gaps through collaborative practice.

The elective embeds PGY-5 psychiatry residents within the clinic longitudinally for 1 − 2 days per week for 12 months. Residents provide psychiatric management, including comprehensive psychiatric assessment and development of interdisciplinary treatment plans, with opportunity to collaborate longitudinally with referring MD providers (family doctors, infectious disease specialists), community mental health workers and caseworkers, and other community agencies. The residents collaborate as part of the interdisciplinary team in the clinic and as part of informal teams with community service providers. The residents receive regular feedback from observed encounters and supervision with psychiatrists in the clinic, with special attention to challenges in providing mental health care for complex populations, developing an approach to care of complex patients, engaging formal and informal teams to advance care, and reflexivity about their own experience providing care while navigating diagnostic ambiguity. Teachers were instructed to focus their teaching on integrated instruction (providing explanations for *why*) and development of integrative competencies (Table 1) that combine intrinsic roles of the physician, emphasizing collaborator, communicator and health advocate roles.

**Table 1:** Competency-based Learning Objectives Utilizing a CanMEDS Framework, with selected examples of specific competencies. Overarching philosophy of care: patient-centred care for this complex population (those diagnosed with HIV, mental illness, and other forms of psychosocial complexity) is achieved through representation of diverse perspectives – from patients and invested care providers – and through collaboration formalized through shared learning and work opportunities

Competency Domain	Specific Competencies	CanMEDS competencies
Comprehensive understanding of complexity for individuals with triple diagnosis	Recognize how syndemics of triple diagnosis amplify comorbidities and negatively affect health outcomes.	- Medical expert - Health advocate - Communicator
Integrated medical and neuropsychiatric assessment and management of patients	Conduct comprehensive psychiatric assessment of individuals with triple diagnosis, including evaluation of common neuropsychiatric findings (i.e., basic neurological exam, cognitive screening), gathering collateral information, and be able to recognize indication for further investigations (i.e., labs, imaging, formal neurocognitive testing).	- Medical expert - Communicator
Psychotherapeutic skills as a means for engagement and traversing barriers to health for this complex population	Demonstrate an appreciation of patient lived experience, and a non-judgmental approach to eliciting patient narrative through clinical interview, recognizing the impact of trauma and stigma on assessment process. Support patient’s active role in treatment and empower patient’s knowledge of their own strengths and challenges to guide therapeutic course.	- Communicator - Health advocate - Scholar
Navigation of systems of care across community and hospital systems, including interdisciplinary models of care for individuals with triple diagnosis	Recognize patient’s experiences of disjointed systems of care, limited mental health resources, and the interaction between the above and other social determinants of health acting as barriers to care.	- Health advocate - Leader

### Participants

Purposive sampling method was used in this study, allowing for focus on specific participant populations who were involved in the resident elective in the clinic. Study participants included: (a) residents doing an elective in HIV psychiatry; and (b) clinicians who work in the clinic, including seven psychiatrists, an occupational therapist, and a social worker. They were recruited through email to participate in the interviews.

### Data collection

Ethics approval for this research was granted by the Research Ethics Board at Sinai Health in Toronto, Canada. Data collection commenced December 2019 with the initiation of the resident elective and is ongoing. All residents and clinicians were interviewed at the beginning and end of the elective. Interviewers were not involved in resident education or assessment. All participants provided written or verbal informed consent for the interviews. Data analysis was conducted simultaneous to data collection, allowing themes identified during data analysis to inform data collection. Residents and clinicians were asked about their experiences providing care in the clinic to those with HIV and mental illness, working collaboratively, and about the resident elective. All interviews were recorded and professionally transcribed by an outside company.

### Data analysis

Thematic analysis [19] of the interview transcripts was carried out by DC and SG. This manuscript describes secondary analysis of a subset of the data from a broader education research study. Qualitative research occurs in context and is influenced by perspective. DC is a consultation/liaison psychiatrist, and the Associate Program Director of the Psychiatry Residency Program at the University of Toronto. SG is a research coordinator in the department of Psychiatry at Sinai Health, with expertise in qualitative research and thematic analysis. RM is the head of research for Psychiatry at Sinai Health, with expertise in attachment research, including of medically complex patients. MM is a senior scientist at The Wilson Centre, with expertise in qualitative research and adaptive expertise. Employing a constructivist analytical framework, interview data were iteratively coded through the generation of open codes. Using NVivo (Version 12, QSR International Pty Ltd., Victoria, Australia), these codes were then grouped into different categories and sub-categories, capturing themes that were identified using a deductive and inductive approach [20]. In weekly meetings, codes were interpreted and analytical frameworks were proposed, drawing on literature about adaptive expertise. Through separate bi-weekly meetings with RM and MM, the thematic structure and analytic framework were further refined and supporting quotations selected. This process occurred from October 2020 to September 2022 and allowed new data to be compared with existing data, resulting in an iterative comparative approach. An audit trail was maintained throughout.

## Results

Twenty-four qualitative interviews with 12 participants were carried out from December 2019 to September 2022, contributing to the findings below. Three residents, seven psychiatrists, one occupational therapist and one social worker completed pre- and post-interviews. Interviews ranged in length from 40 − 70 min, leading to transcripts 10 − 24 pages in length.

The results of the thematic analysis elucidated the contextual definition of complexity for this patient population, which informed the trainees’ conception of an approach to caring for patients with complexity.

### Encountering complexity in daily clinical work

Prior to the elective, residents appreciated that the most complex patient populations have multiple problems, including psychiatric, medical and psychosocial comorbidities, and sought out this elective to learn more about complexity management.

Patient complexity was a key focus of learning for the residents, recognizing,

just how complex people can be. How really sick they can be. Medical illness, psychiatric illness, substance use, poverty, marginalization, racialization. Lots of challenges people are facing. (Resident 2)

Trauma was also prevalent:

the amount of trauma that some of the patients had was quite different from my previous experiences. That I was not as prepared for. (Resident 1)

Residents noted that trauma could derive from experiences of social oppression:

Traumatic experiences over the course of childhood were quite common. Maybe they wouldn’t meet criteria for what the DSM defines as a single traumatic instance, but certainly the discrimination and the marginalization around race or sexual orientation or gender identity. All of those kinds of things building up over time [and] was obviously quite common with most of the people that I was seeing in this elective. (Resident 3)

To equip trainees for this complexity, supervisors emphasized the importance of having an approach:

my agenda for teaching the trainees is to help illustrate why this is a unique population to treat and how having some clear approaches to complexity… and intersectionality is important in psychiatry, and how HIV psychiatry can be a model for that. (Staff 2)

Residents described improved understanding of the syndemics of HIV:

I’ve learned to fine-tune my ability to recognize the interactions between HIV, substance use, and mental illness, and how they’re all intrinsically linked. It’s … important to have an understanding of these challenges before you can treat it, an understanding that they’re all connected. It helps to be a clinician that’s addressing the whole person, rather than just the substance use physician or the psychiatrist. (Resident 2)

After the elective, residents shared that their prior experiences of complex patients were often in acute medical or psychiatric inpatient settings or in brief consultation models. Residents had less experience providing longitudinal care to patients as complex. The elective experience enriched trainee appreciation of the diverse impact of adverse childhood experiences (ACE) and trauma, compounded by other intersectional factors (immigration status, gender and sexual identity, experiences of structural racism and stigma), on diverse aspects of care, including engagement, accurate assessment and diagnosis, implementation of treatment plans, and outcomes. The ability to identify and describe factors that contribute to complexity is an important first step toward development of adaptive expert capabilities.

### Developing an approach to complexity: inviting uncertainty

Trainees and supervisors said that one of the teaching goals of this elective was the importance of normalizing “not knowing”, and of inviting uncertainty when the diagnosis may not be obvious upon initial assessment. There was also acknowledgement that “patient complexity” reflects as much the system as it does the patient. A supervising psychiatrist remarked,

humility is one of the responses to complexity. It’s a moving target, you … have to try and stay present and tolerate ambiguity. To me, that’s a sign of a mature clinician, that they can tolerate the ambiguity and still accompany that patient during that ambiguity. (Staff 7)

Another supervising psychiatrist taught:

an approach to complexity, … and an understanding of the variables that go into complexity. An understanding of where we fit into that whole scheme of complexity, … what our actual capacity is, where there’s room for capacity building and our limits. And where we need to become more interdependent with other agencies to fulfil those needs. (Staff 5)

Residents described an ingrained urgency to complete an assessment within the initial meeting with a patient, and they perceived uncertainty as a personal failing. They reflected that normalizing and making explicit this experience of uncertainty while chartering the care of complex patients was important for patient care and to prevent burnout.

The residents discussed how they became comfortable with complexity:

After the elective, there was better comfort [assessing] patients with multiple complaints. HIV Psychiatry, … it’s tougher from a variety of biopsychosocial perspectives because of the biological impacts of the infection, and the psychosocial stigma that comes with it. In residency, … we’re told to do 50-minute assessments…. [It’s about] being comfortable with [taking] longer, and sort[ing] through things. It gave me more confidence [and] flexibility. (Resident 1)

The residents also spoke about developing comfort with ambiguity and the limitations of the physician role:

I’m coming to the realization, as my medical training goes on, that oftentimes there isn’t one right answer, … there might be multiple right answers. As long as you are taking some degree of an evidence-based approach, working with compassion, working with the patient, and being honest about your own limitations and concerns—that really helps to navigate ambiguity. (Resident 3)

That sentiment is echoed by another resident:

You’re never going to be perfect and just accept that … At the end of the day, … the most important thing you can do is support somebody and be there, … not abandon patients when they’re ill…. We can get frustrated when things aren’t working…. We can feel inadequate or like a bad doctor when really it’s just a complex situation. (Resident 2)

### Harnessing integrative competencies to navigate ambiguity

In addition to content or contextual knowledge, residents described integrative competencies – competencies that combine multiple essential roles of the physician (i.e., medical expert, collaborator, communicator, leader, etc.) that they gained through the elective. Residents had opportunities to expand medical knowledge in specific content areas: neuropsychiatry of HIV, pharmacology for patients with triple diagnosis (HIV, SUD, mental illness), and cognitive testing. They also had opportunities to strengthen skills for assessment, diagnosis, and engagement in care. Participants observed the importance of integrative competencies, especially when caring for patients with significant trauma histories where trauma was not the presenting concern, yet a trauma-informed approach in combination with other competencies was essential for clarifying assessment:

the amount of trauma that some patients had was quite different from my previous experiences…. The model of care here is different from [other] sites … where it was more of a medical model. That didn’t prepare me … to work with these folks…. I think I developed some more skills, comfort.… I can think of two cases where the experience of trauma and abuse was so much. From a medical perspective, that would be missed. I think supervision here allowed me to grow with that. (Resident 1)

Importantly, participants spoke about consolidating knowledge and skills through collaboration with an interdisciplinary team (within the clinic, and beyond through the circle of care). Residents described grappling with potentially conflicting perspectives (i.e., neuropsychiatric and psychodynamic perspectives, the primary care and infectious disease perspectives, or the community case worker’s perspective on how the patient is living day to day), learning to include these in a formulation, and using this to clarify next steps for management. A supervisor said:

Hearing from other people with different levels of experience could be helpful, and any opportunity to speak to the uncertainty is helpful in coping with it. (Staff 3)

The residents discussed the benefits of having a collaborative, multidisciplinary case conference in the HIV Psychiatry clinic:

… everyone had a different perspective, which was helpful to absorb…. Everyone had a different lens, and that’s not easy to find. It’s such a valuable experience that people devote time to provide their input. (Resident 1)

Residents described working with social workers within the clinic, or with community case workers, and benefiting from both the concrete supports they could provide patients and learning from the differences in approach – between hospital and community-based work, and from a different training perspective. Reflecting on their experience working with a community mental health clinician, a resident reflected:

Sometimes we would have differing opinions, which was great, understanding other opinions … gives a richer formulation of the patient. (Resident 2)

Residents also reflected on how collaboration beyond the clinic made them aware of their role in sustainably caring for complex populations:

as a psychiatrist, your role is first and foremost a consultant to your referring doctor, so whether it’s a family doctor, NP, infectious disease doctor, … really supporting them in what they do in the community. (Resident 2)

### Developing an approach to complexity through adaptive expertise

The residents described improved confidence in tolerating uncertainty and engaging innovative approaches to patient complexity. This approach aligns with understanding of adaptive expert performance [18, 21]:

it’s such a nuanced thing that everyone has a different approach to complexity…. You have to absorb it from different people and come up with your own way … of approaching complexity. (Resident 1)

Later this resident explained:

there’s not always a perfect solution, there’s no perfect answer. There’s a blatantly wrong approach, and there’s a blatantly wrong management…. When things are complex, many options can be right, and we have to tolerate that uncertainty. (Resident 1)

As residents developed confidence in their approach to complexity, the ability to improvise when necessary became evident:

we’re taught a specific way to do a psychiatric interview, starting with mental health issues and a review of mental health symptoms, then talk about substance use and … medical history. I found it quite helpful to switch that up a bit and start with a social and personal history, and just take some time at the beginning of the interview to get to know the person and to show that you have a genuine interest in who they are and their life experiences. I think that was often the first sign of some trust within my relationship with the patients (Resident 3)

This shift toward improvisation shaped the residents’ entire approach to care:

particularly people who are marginalized, … you need to be able to accept people where they are, and flexibility is also very important. You can’t be a rigid clinician in these environments. Being able to turn on a dime, reschedule, switch up your care plan depending on where the patient is at is important. (Resident 2)

Furthermore, the residents were able to combine conceptual (why) and procedural (what) knowledge during the elective experience, which is evidence of adaptive expertise:

the diagnosis of HIV is your marker of marginalization for a lot of people. It means that maybe they haven’t had access to prophylactic care or medical care … or they were assaulted or … their substance use illness is dangerous. If you’re not thinking about these things, you’re really missing the boat on providing care for people with HIV…. The elective has really helped to … give me the opportunity to think about these things with the patient in front of me. Take it from a theoretical to a practical. (Resident 2)

This act of taking the “theoretical to a practical”, understanding the *why*, and integrating this into a management plan, is a feature of adaptive expertise; as is the ability to draw on and balance conceptual and procedural knowledge while adapting to new contexts and the varying needs of patients [18, 21–24]. Further, we observed residents become more flexible with themselves, and integrate self-compassion in response to “not knowing” within the learning and practice environment. They described this as protective from burnout when encountering complexity.

## Discussion

Physicians feel ill-equipped managing complex patient cases [3, 11, 12]. The aim of the HIV Psychiatry elective described here was to foster adaptive expert capabilities for approaching the care of complex patients, such as those with HIV, mental illness, and other psychosocial barriers to health. This study elucidates certain competencies that empower physicians to care for complex patients: the ability to invite uncertainty, learn and integrate different kinds of knowledge, navigate and devise a treatment plan when there is diagnostic ambiguity; also, to collaborate and learn from an interprofessional team environment across hospital and community settings. This preliminary work done in a psychiatric setting highlights that trainees can feel more competent, confident and engaged in caring for complex patients when an approach to “not knowing” is made explicit and integrative competencies that emphasize collaboration are seen as essential.

Beyond the psychiatric setting, collaborative competencies can help physicians to fill in their own knowledge and practice gaps, while critically thinking about an approach to care when evidence-based standardized approaches fail. Residents in our study changed their perspective on “not knowing,” and learned that the solution for a complex patient problem required reaching out to other providers, acquiring more information, and helping patients navigate a complex system. Explicit acknowledgement by teachers of the experience of uncertainty in response to ambiguous and complex encounters was an important starting point. Residents and staff reflected that this stance of epistemic humility, and a collaborative approach that recruited a team, was also protective from feeling burnout, which most cited as a real risk of caring for complex populations.

Adaptive expertise is the capacity to perform routine tasks, drawing on past knowledge and experience, alongside the ability to flexibly shift when necessary to address novelty and uncertainty [22, 23, 25]. Working collaboratively with other providers can improve this improvisation and flexibility [13, 23]. The elective residents described an integration of theoretical and practical knowledge, and a greater ability to flexibly engage with patients by meeting patients where they were – key characteristics of adaptive expertise. The residents also applied critical thinking and collaborative skills of an adaptive expert learner, which allowed them to support complex patients to obtain the care that they needed, even if the diagnosis was not obvious from the start [13, 23, 24].

Our study has limitations. Firstly, these results are preliminary, based on the first three years of the elective offering. Therefore, more study is needed, and in broader clinical contexts. Secondly, all participants were from Toronto, Canada, so consequently, our findings may highlight issues specific to our region. Thirdly, participants selected the experience, and may reflect a group already interested in and primed for this challenge. If clinical experiences like this were required, calibration of the experience in a more general way may be necessary.

## Conclusion

We argue that clinical experiences that deliberately teach an approach to uncertainty, normalize “not knowing”, and model integrative competencies that emphasize collaboration, help to hone adaptive expertise. It is well known that care of complex patients best occurs in collaborative models [11], and our teaching and education should reflect this. Moreover, the way we collaborate is important. This study highlights the importance of modelled humility, and the learning that occurs from exchanging perspectives and not simply sharing tasks as a multidisciplinary team. With each complex patient presenting in unique ways, the development of epistemic humility, flexibility and improvisation – key characteristics of adaptive expertise – are necessary to competently care for complex patient populations. This adaptive expert approach to clinical work may also foster provider resilience. Participants in our study described an ingrained culture of “knowing” in medicine and medical education, that when explicitly challenged, more nimbly allowed providers to effectively recruit support in navigating their uncertainty in service of their patients. Physician burnout is a complex issue, primarily related to the stressors present in the work environment, and the way we teach in diverse contexts needs to incorporate this nuance.

We are currently in the process of piloting a fourth year of the resident elective experience, with the aim to further explore the significance of these findings. Going forward, findings from the study will be used to inform this ongoing elective.

## Data Availability

The datasets used and analyzed during the current study are available from the corresponding author on reasonable request.
